# Disconnected relationships between primary care and community-based health and social services and system navigation for older adults: a qualitative descriptive study

**DOI:** 10.1186/s12875-020-01143-8

**Published:** 2020-04-23

**Authors:** Ruta Valaitis, Laura Cleghorn, Jenny Ploeg, Cathy Risdon, Derelie Mangin, Lisa Dolovich, Gina Agarwal, Doug Oliver, Jessica Gaber, Harjit Chung

**Affiliations:** 1 Aging Community and Health Research Unit, School of Nursing, McMaster University, HSC 3N25, 1280 Main Street West, Hamilton, ON L8S4K1 Canada; 2 Department of Family Medicine, McMaster University, David Braley Health Sciences Centre, 100 Main Street West, 5th floor, Hamilton, ON L8P 1H6 Canada; 3 School of Nursing, McMaster University, HSC 3N25, 1280 Main Street West, Hamilton, ON L8S4K1 Canada

**Keywords:** Primary care, Social care, System navigation, Community services, Older adults, Multiple chronic conditions, Health and social services

## Abstract

**Background:**

There are gaps in knowledge and understanding about the relationships between primary care and community-based health and social services in the context of healthy aging at home and system navigation. This study examined provider perspectives on: a) older adults’ health and social needs; b) barriers to accessing services; c) the nature of relationships between primary care and health and social services; and d) ways to facilitate primary care and health and social services navigation to optimize older adults’ health.

**Methods:**

Four focus groups were conducted involving providers (*n* = 21) in: urban primary care clinics and health and social services organizations serving older adults in Hamilton, Ontario, Canada. Purposive sampling was employed to recruit community health and social services managers, directors or supervisors and primary health care providers in a Family Health Team via email.

**Results:**

Health and social services needs were exacerbated for community-dwelling older adults with multiple chronic conditions. Strong family/caregiver social support and advocacy was often lacking. Access barriers for older adults included: financial challenges; lack of accessible transportation; wait times and eligibility criteria; and lack of programs to address older adults’ needs. Having multiple providers meant that assessments among providers and older adults resulted in contradictory care pathways. Primary care and health and social services linkages were deficient and complicated by poor communication with patients and health literacy barriers. Primary care had stronger links with other health services than with community-based health and social services; primary care providers were frustrated by the complex nature of health and social services navigation; and care coordination was problematic. Health and social services referred older adults to primary care for medical needs and gathered patient information to gauge program eligibility, but not without challenges.

**Conclusions:**

Results point to strategies to strengthen primary care and health and social services system navigation for older adults including: using a person-focused approach; employing effective primary care and health and social services communication strategies; applying effective system navigation; building trust between primary care and health and social services providers; advocating for improved program access; and adapting services/programs to address access barriers and meet older adults’ needs.

## Background

Globally, the proportion of older adults is increasing as is the proportion of older adults living with multiple chronic conditions (MCC) (defined as two or more chronic conditions) [1]. Older community-dwelling adults can experience significant challenges in managing their health, especially if they also experience long-term social conditions (e.g., poverty, poor housing), which add to their care needs [2]. Primary care is typically the first point of access for managing the health of older adults, which places heavy demands on providers. Referrals that link people receiving health care to community services and the voluntary sector to address social conditions tend to originate from primary care [2]. However, a international survey of older adults showed that Canada had some of the poorest results in care coordination that involved linking with social service providers [3].

Older adults, their caregivers and providers have found that managing MCC is overwhelming, draining, and complicated; it involves frequent challenges in getting help such as community support services necessitating reliance on family and friends [4]. Holistic care, particularly for those with MCC requires attention to address integrated health and social care needs [5]. Integrating health and social services for older adults can increase their capacity to manage chronic conditions [6, 7].

In 2016, primary care physicians at an international World Organization of Family Doctors (WONCA) conference, developed five ‘rules’, or operating principles, for multimorbidity care, one of which was to “engage with the community to enhance social networks and health-promoting infrastructures” [5] p. 117. Improving linkages between primary care and community-based health and social services (HSS) can involve multiple activities (e.g., information provision, making referrals, and facilitation) to improve patients’ access to health care and community-based resources to improve their health and well-being [2]. In this paper, the HSS sector refers to a broad-based group of voluntary and community organizations that provides programs and services to address well-being (e.g., senior’s centres), health needs (e.g., nurse home visiting programs), as well as social care needs (e.g., transportation, caregiver and home support). Older adults experience barriers in navigating the complex system of primary care and HSS, and this requires support. A scoping literature review of system navigation in primary care indicated that the role is assumed most often by a designated navigator (a paid professional or trained lay person) or it is shared by a team through assigned navigation functions [8]. Effective linking of older adults and their caregivers to HSS, also known as “social prescribing”, requires expertise in system navigation [9]. System navigation involves facilitating access and reducing barriers to services as well as facilitating continuity of care which can result in more effective and efficient health and social services systems use [8]. In the effort to improve health outcomes for vulnerable populations such as older adults, those living with mental illness and to combat social determinants of health such as social isolation, social prescribing appears to have some benefit to patients’ experiences and access to services [9]. The evidence is weak however, to assess their impact on health outcomes, or to identify models and components of successful interventions [9]. Older adults experience barriers in navigating the complex system of primary care and HSS requiring support. More research is required to understand the nature of linkages between primary care and social care, the tools or mechanisms to facilitate linkages, and what else is required to reduce barriers to system navigation. Successfully addressing coordination and linkages between primary care and HSS for the provision of seamless care requires coordinated efforts by all sectors.

Strengthening integration and linkages to support system navigation across health care and HSS has been a key health care issue over the last decade in Canada [10–16]. Other nations, such as the UK, have developed policies to transform care through integration between health and social care [17, 18]. However, primary care and other health care organizations do not often link their patients with HSS due to a lack of awareness and poor access [2, 11, 18–20]. There is a paucity of research that deeply explores the nature of this intersectoral relationship from the perspectives of both primary care as well as HSS providers, and how these relationships have been or could be nurtured in the context of addressing older adults’ needs and gaps in services. Therefore, the overall aim of this paper is to understand how primary care and HSS providers perceive the health and social services needs of community-dwelling older adults and how they work together to address their barriers in accessing services to address those needs.

The following research questions address these knowledge gaps:

1. What do primary care and HSS providers perceive are common health and social service needs, barriers in accessing primary care and HSS, and service gaps experienced by community-dwelling older adults?
2. What is the nature of relationships between primary care and HSS?
3. What system navigation strategies do primary care and HSS providers use to support older adults to access services?

## Methods

This qualitative descriptive study [21] involved four focus groups that were conducted in 2014 in Hamilton, Ontario, Canada. Two focus groups were held with HSS managers and coordinators that serve older adults. In addition, two focus groups were held with providers (e.g., family physicians, nurse practitioners, pharmacists) working in two primary care clinics under a Family Health Team model. The Family Health Team model was implemented in Ontario in 2005 [22]. It applies the principles of a patient-centred medical home and provides multidisciplinary team-based care and access 7 days per week. Family Health Teams are supported by a blended model of funding (capitation, fees for services, bonuses for reaching targets, and payments for specialized programs) [22]. A purposive sampling strategy was employed to recruit front line providers and managers working in Hamilton, Ontario as this site was to be involved in a future intervention study involving system navigation as a component of the intervention [23]. Participants were eligible if they were primary health care providers in a Family Health Team or were managers, directors, or supervisors in a community-based organization providing home and community support to older adults. Community service providers were identified by the research team for recruitment to obtain a diverse sample of common types of services provided to seniors such as: housing, home health care, case management, home support, and disease management (i.e., diabetes). Primary care team members were identified from the academic Family Health Team in the city. Participants were recruited via email invitation by the research team.

Two female facilitators experienced in qualitative research [one PhD prepared nursing faculty member (RV) and a Masters prepared research coordinator (LC)] conducted focus groups with one leader and two note-takers present. Brief field notes were taken to note non-verbal cues and general observations. Focus groups (see supplementary material for the focus group guide) were conducted at the two Family Health Team clinic conference rooms and in an off campus university building. RV and LC, who were working in or associated with the Department of Family Medicine, had some familiarity with a few primary care participants since the Family Health Team is affiliated with the department. Participants were informed at the start of the interview that the research team was interested in learning about participants’ experiences with and perceptions of older adults’ needs and their linkages between primary care and HSS to inform the design of future interventions to support system navigation. Examples of interview questions were: “What do you find are the most challenging health and social service concerns/needs to address for older adults living in the community?” and “What strategies/mechanisms do you and your organization use to build and sustain credible linkages with HSS/ primary care to support the health and well-being of older adults living in the community?” These broad guiding questions were not provided to participants ahead of time, nor were they pre-tested. No follow up interviews were conducted nor were transcripts or results shared directly with participants. Focus groups lasted from 45 to 80 min, were audio-taped and transcribed verbatim. Participants received an incentive for participation ($25 CAD gift card) and refreshments.

Thematic content analysis of transcripts was conducted using NVivo 10 applying a descriptive qualitative approach [21]. Interview questions provided the initial organizing framework (coding tree) for text that was inductively coded. Two coders reviewed the coding structure after coding one transcript (RV, LC). Once agreement was reached on the approach, remaining transcripts were coded by LC, RV and HC (two experienced qualitative researchers, and an undergraduate nursing student). Data were collapsed into themes by LC and HC, refined and confirmed by RV using constant comparison. Themes were reviewed by JP to confirm that they were supported by data (quotes). The full research team consisted of nursing and health sciences researchers, a pharmacist, and primary care physicians. They validated the final themes and quotes to enhance study credibility and rigor. Investigator triangulation supported the analysis and dependability and credibility of results. An audit trail was maintained of data interpretation and all decisions.

## Results

Table 1 reports study participant characteristics in detail (*n* = 21). Twelve interdisciplinary primary care providers and nine HSS providers from organizations serving older adults participated. Table 2 provides a summary of themes (presented in italics in the text) and sub-themes, where relevant, for each research question. Quotes in the text to support each theme are distinguished by: the type of respondent (HSS or PC [primary care]), focus group (1 or 2), and participant ID (e.g., [HSS1–1], [PC2–1]).

**Table 1:** Characteristics of Study Participants (*n* = 21)

Characteristics	n
**Participants (*n* = 21)**	
Primary Care Providers	12
CBHSS Providers	9
**Location of Primary Care Providers (*n* = 12)**	
Clinic Site 1	5
Clinic Site 2	7
**Primary Care Service Providers’ Discipline (*n* = 12)**	
Nurse Practitioner	4
Physician	2
Pharmacist	2
Dietician	1
Occupational Therapist	1
Registered Nurse	1
Social service worker	1
**Community Service Providers’ Role (*n* = 9)**	
Coordinator	4
Case Manager	2
Director	2
Supervisor	1
**Organization Type of Community Service Providers (*n* = 9)**	
Seniors’ case management and day programs	3
Seniors’ housing and recreation	2
Seniors’ recreation and day programs	1
Home care	1
Home support services (e.g., housekeeping)	1
Diabetes-related programs and services	1

**Table 2:** Themes and Sub-themes by Research Question

**Research Question 1: PC and HSS Providers’ Perceptions of Common Health and Social Service Needs, Barriers in Accessing PC and HSS, and Service Gaps Experienced by Community-dwelling Older Adults**	
HHS Needs of Older Adults	
• Need for improved management of multiple chronic conditions • Need for a shared understanding of older adults’ needs and abilities • Need for understanding and acceptance of services and supports • Need for social support and advocacy	
Barriers to Accessing HSS and PC Services for Older Adults	
• Untimely access • Restrictive eligibility criteria • Financial challenges	
Service Gaps for Older Adults	
• Lack of affordable and accessible transportation • Challenges with communication • Health literacy	
**Research Question 2: The Nature of Relationships between PC and HSS**	
• HSS struggling to communicate with PC • PC has stronger linkages with core health services than with HSS • Frustration with the complex nature of HSS system navigation • Poor care coordination	
**Research Question 3: System Navigation Strategies to Facilitate Access to Services for Older Adults**	
**Theme**	**Sub-theme**
• Improving partnerships between service providers	• Employing effective communication strategies • Building trust and rapport between HSS and PC
• Working to meet the needs of the client	• Using a person-centred approach • Applying effective case coordination • Adapting services/programs to address access barriers and fill gaps for older adults’ needs • Advocating for improved program access for older adults

### Research question 1: needs, barriers, and service gaps for older adults

Participants focussed on older adults with the greatest health and social needs; that is, older adults living with MCCs. Four themes were identified that reflect the most common health and social service needs of community-dwelling older adults. Participants agreed that older adults had *the need for improved support for self-management* to manage multiple chronic conditions (e.g., hypertension, diabetes, dementia). Improving self-management involved addressing medication adherence; nutrition concerns; and management of functional issues, such as sensory or mobility impairments. Multiple and frequent specialist appointments added further complexity and contributed to a lack of comprehensive care and challenges for providers:

“It feels like a daily game of Tetris. You’re adjusting all the components to fit properly.” [PC1–7]

Mental health issues or cognitive decline also intensified challenges in self-management support. Often crisis management rather than prevention took priority in the provision of care. The intensity of needs, frequent health status changes, and crises made a pro-active approach to self-management challenging for patients, families and providers.

“ … It’s often almost a little too late.” [HSS1-3].

All participants agreed that there was *a need for shared understandings of older adults’ needs and abilities.* Participants raised the problem of incongruent assessments of older adults’ needs among providers, patients, and their family caregivers and older adults and families were unable to recognize gradual health decline:

“The family doesn’t see it when [declining health] is gradual. They only see it when it’s grandiose, and it’s a problem at the end.” [PC2-unidentified participant]

Providers’ also perceived that some patients and family members were in denial, signaled by the refusal of help which related to the theme - *the need for understanding and acceptance of services and supports to address older adults’ needs*. Some participants viewed older adults’ resistance to help was related to low self-efficacy, doubting older adults’ ability “to make changes” [PC2–3] or seeing them as “set in their ways” [HSS2–3]. Participants wanted to reach a shared understanding of patients’ needs within the circle of care and older adults’ acceptance of available services to address them.

Finally, the *need for social support and advocacy from nearby family or friends* was deemed essential. All provider groups highlighted the need for someone to accompany older adults to appointments to be their advocate. Older adults struggled with instrumental activities of daily living (e.g., meal preparation, housekeeping) which affected older adults’ ability to age in place and resulted in missed opportunities to implement needed supports:

“A lot of my clients don’t have support of family members or friends, aside from us being involved. And even if they do make it [to their appointments], they don’t have that second set of ears and eyes to really pay attention and grasp what’s being said...” [HSS2–1]

Three themes related to barriers to accessing HSS and primary care health services. All focus groups raised concerns related to *untimely access*. Poor access to primary care physicians raised anxieties when health issues were escalating. Some patients experienced restrictions by physicians to allow only “one [health] issue at a time” [HSS2–4] to be discussed at each visit. This was incredibly frustrating for older adults living with MCC which added to untimely management of their needs. *Complex and restrictive eligibility criteria* was another theme. Wait lists to obtain services and strict criteria to be eligible to receive services (e.g., having friends or family to help limited eligibility to receive home support services) created barriers to access. Furthermore, bureaucratic paperwork such as “waivers and legalities and confidentiality” [HSS2–3] were additional barriers to access HSS. Older adults’ *financial challenges* were raised in all focus groups as an access barrier contributing to social isolation. Poverty forced older adults to make difficult choices:

“ … if I’m telling them [ … ] to do a diabetic follow up. Well they need blood work. ‘Well which one do you want me to do? Either I come to see you, or I have enough money to go to get the blood work.’” [PC1–7]

Three themes related to service gaps for older adults. Both groups noted a *lack of affordable and accessible transportation* that was related to unreliable and inconvenient transit services, late schedules, transportation and parking costs, and physical accessibility challenges. They also noted access gaps related to *a lack of attention to older adults’ communication challenges* and *health literacy.* Challenges experienced by non-English speaking older adults and the ‘language of health’ (i.e., health literacy) were issues to be considered to ensure that health information is communicated and understood. Communication barriers were exacerbated by challenges using phone services:

“ … they often ask us to call because they just don’t feel confident around the terminology that people will be using on the phone.” [HSS2–5]

Both groups agreed that an advocate was key to supporting effective communication.

### Research question 2: nature of relationships between primary care and HSS

The nature of relationships between primary care and HSS was also explored. Overall, strong primary care and HSS relationships were rare. Four themes illustrate the nature of these relationships: (a) *HSS struggles to communicate with primary care*; (b) *primary care has stronger linkages with core health services than with HSS*; (c) *frustration with the complex nature of HSS system navigation*; and (d) *poor care coordination*.

HSS providers spoke extensively about *struggles to communicate with primary care*. They typically were able to gather limited information, such as a physician’s contact information, or confirm client eligibility for a HSS program:

“The only time we ever talk about or address the real medical issues are when [the patient] may not qualify under the seniors’ component for the program and they have to show that they have a permanent disability.” [HSS2–3]

Obtaining consent to exchange primary care’s information was another challenge. Primary care participants confirmed that they infrequently referred patients to HSS directly but provided assessments for HSS to determine program eligibility. Shifting eligibility criteria for HSS programs added barriers to this process.

Primary care providers reported having much *stronger linkages with core health services* (e.g., speciality care, emergency care, home care, and pharmacies) *than with HSS*. HSS providers agreed:

“It’s very infrequent too where a medical office will call and get information on the [HSS] program, which I think is very proactive. They must have the client that is looking … ” [HSS2–3]

Primary care typically connected with HSS through a primary care-based system navigator or regional home care coordinator. They tended to contact HSS if they had a past working relationship, or in response to patient requests in relation to access barriers (e.g., housing). Primary care providers expressed the need to determine the credibility of HSS before making a referral. Weak linkages were largely due to a lack of trust and understanding of HSS. HSS providers validated this:

“ … [primary care are] looking at a pamphlet, they just don’t get enough understanding or they don’t feel comfortable because they feel responsible. If they’re a physician or a nurse, and they’re sending you to a service, they want to make sure they’re sending you to a credible, reliable service.” [Interviewer summarizing the discussion in HSS2]

The need for trust was greater when dealing with older adults who are considered vulnerable.

Both provider groups expressed frustration with *the time consuming, complex nature of HSS navigation,* due to the number of health and social service agencies, and little coordination between them. Primary care providers particularly found system navigation with HSS overwhelming, time consuming, and lacked time to learn about their services, although they wanted to learn more:

“I would love to take a few days to go out and meet all these services, and find out about them. That would never fit into my schedule.” [PC1–6]

Another challenge contributing to poor familiarity with HSS was the lack of continuity in HSS providers and constantly shifting program offerings:

“Often, I find community agencies make changes because they have to. Their funding has changed [ … ] but they don’t always communicate out to the stakeholders that are going to be impacted by those changes.” [PC2–3]

A few primary care providers questioned their role in relation to system navigation, particularly given the time it takes to build HSS relationships:

“We don’t have the time to be able to do that [system navigation],” and “even though it’s not truly our job, we do it … ” [PC1–4]

Providers’ lack of knowledge of HSS services to address older adults’ needs contributed significantly to *poor care coordination* and inefficiencies:

“You’re doing your networking, and you’re thinking ‘God, if I’d only known that I would have saved ten calls.” [PC1-ID4]

Further, both groups identified how receiving care from multiple primary care and HSS providers added complexity to care coordination:

“You get [patients] to somewhere, and then in some ways, you lose connection with them. But they come back here when there’s something going wrong. And you’re not always sure what has gone on, why it went off the rails.” [PC2–3]

### Research questions 3: strategies used to address needs of older adults

Participants identified two themes related to system navigation strategies to facilitate access to primary care and HSS programs and services for older adults: *improving partnerships between service providers* and *working to meet the needs of the client*. Table 2 presents these themes and their sub-themes.

*Improving partnerships between service providers* included the sub-theme: employing effective communication strategies. It was raised by all provider groups, but more frequently by HSS. Some spoke about trying to develop more partnerships with community agencies to enable referrals and build better connections with health care.

“[We are] trying to make connections with [primary care teams] and who’s the most appropriate within that structure to offer information; that will be of benefit to clients.” [HSS1–3]

HSS providers also spoke about accompanying older adults to primary care appointments to increase awareness of HSS services as well as approaching the most appropriate providers who could promote their services. Some gained primary care access through nurse practitioners’ or family practice nurses’ previous connections. However, HSS providers were often blocked from connecting with clinicians by reception staff, leading some providers to work to build stronger relationships with receptionists.

Another sub-theme - building trust and rapport between HSS and primary care - was raised in most focus groups, to improve system navigation. Primary care providers identified that building trust and rapport required increasing their knowledge and awareness of, as well as connections to, HSS programs and services. A few noted that this was best done by: getting older adults’ feedback or endorsements based on their experiences; sharing providers' consult notes; or having work experiences in other settings.

“I worked as a visiting nurse in this community, for about twenty years. So over those twenty years, prior to becoming a nurse practitioner I developed lots of relationships [with HSS]”; [PC2-4]

Having trusting relationships could help to sustain provider networks.

In the next theme - *working to meet the needs of the client-* the sub-theme, using a person-centred approach, was the most reported strategy. Participants felt strongly that programs and services need to meet older adults’ needs. This entailed building rapport and using effective communication strategies with older adults, demonstrating real attempts to match patient capacity to available services. It also required providers to respect older adults as individuals and support their right to refuse services. One participant summed it up as follows:

“It’s not about control over who’s doing what; it’s about, really at the end of the day, trying to meet their needs and working together as sort of a circle of care around clients that we have.” [HSS2–4]

Another subtheme, applying effective case coordination, was raised by both provider groups, but mostly by primary care. Primary care providers valued having a care coordinator who knew about HSS resources, such as from the provincial home care program, another team member, or in-house systems navigator. A HSS provider suggested that primary care needs to take more time to comprehensively evaluate situations that arise in the care coordination process, to prevent potential crises from developing.

“[Primary care is] talking about the limited time to support those patients who need a higher level of care coordination. If you were just to, sort of, take that extra time, it would have such a benefit to, you know, stopping crisis a lot of the time.” [HSS-4]

Using case conferences was identified by one participant as an effective way to communicate between sectors.

The sub-theme, adapting services/programs to address access barriers or fill gaps for older adults’ needs, was noted by both groups as a way to support system navigation*.* However, it was manifested in different ways. Primary care providers spoke about reaching older adults through outreach such as house calls, phone visits, or alternate primary care clinics (e.g., shelter health programs). HSS providers described implementing or planning to expand health programs to fill existing program/service gaps.

“If we had people that could come in to do med [ication] reminders or med reviews, people to focus on the physical aspect of health in terms of making sure the people are eating properly [ … ] If you could come out and do some outreach … ” [HSS2–4]

Building a one-stop programs/services shop and conducting a gap analysis to inform new program development were other potential strategies.

Advocating for improved program access for older adults was a less often noted sub-theme raised in all but one HSS focus group. Advocacy was one way to ensure older adults could access HSS and primary care services, although older adults were seen to have challenges in advocating for themselves and need providers to advocate for them. Some noted that providers were dedicated to addressing older adults’ access barriers. One participant noted:

“I’m an advocate for seniors. I work for seniors. I’ve been at it twenty-six years. I’m working for them. Don’t tell me you can’t do it.” [HSS2–1]

## Discussion

This study increases our understanding of providers’ perceptions of older adults’ health and social needs, barriers in accessing services, and service gaps. More importantly, the findings deepen our understanding of the nature of the relationships between primary care and HSS and strategies used by providers to improve linkages between them to better address the gap between older adults’ needs and health and social care services.

Primary care and HSS providers perceived that older adults have significant challenges in managing MCC and need *improved self-management of MCC* and *social support and advocacy.* This is supported by Taylor and colleagues who argue that self-management needs to be tailored to individuals with long-term conditions and supported through a collaborative patient-provider relationship [24]. Our results also showed that current practices result in a lack of continuity in care and communication challenges among older adults and their providers, exacerbating the problem of arriving at shared understandings. Kuluski and colleagues [25] found that goals were often misaligned among older adults, caregivers, and physicians, particularly when patients were less stable. Furthermore, they found that physicians and caregivers were more likely to see the importance of preparing for future decline than did patients. This was thought to be due to triads’ different roles and responsibilities. It is argued that a comprehensive and personalized care plan that defines the patients’ goals, optimizes chronic disease management (including coordination of care that addresses health and non-medical issues) and provides a documented care record may be a key strategy to support a shared understanding of needs and actions to address the needs of complex patients [26].

Participants in our study perceived that older adults are ‘set in their ways’ or have low self-efficacy, and do not accept supports. Ford and colleagues report that health professionals perceive that the inconsistent use of primary care services by older adults is due the gap between their expectations and the adequacy of service provision [27]. Others argue that preferences, goals, and motivational priorities may change in aging, related to adaptation to losses as well as psychological development wherein new viewpoints and roles develop [28]. Taking a person-centred approach to care is critical taking into account older adults’ changing expectations, goals and priorities, and adapting services to meet their expectations rather than expecting their uptake of existing supports.

Both provider groups agreed that older adults face service gaps including a lack of affordable and accessible access to transportation, and a lack of attention to older adults’ communication, and health literacy challenges, which have been reported by others [29, 30]. The World Health Organization’s Report on Aging and Health notes that health service availability implies the extent to which services meet the health needs of older people considering: “non discrimination, physical accessibility, economic accessibility (or affordability), and the accessibility of information” [28]. (p. 14) These dimensions are considered most relevant for older adults with physical limitations, financial insecurity, and challenges with literacy and the use of web-based material [28].

To help address older adults’ needs, barriers and gaps in services requires collaborative efforts from both primary care and HSS sectors. The Agency for Health Quality Multiple Chronic Conditions Research Network Conceptual Model of the Role of Complexity in the Care of Patients with Multiple Chronic Conditions [31] addresses a lack of fit between patient needs and the service system which takes into account health and social needs. It also helps us understand how a “lack of alignment between patient needs and delivery system capacity” creates the needs-services gap [31]. As reported by others [32], our study illuminated weaknesses in primary care and HSS sectors’ capacity and ability to work together, thereby contributing to the needs-services gap. Primary care providers tended to build stronger links within the health care sector than with HSS and lacked trust in HSS. The lack of effective primary care-HSS partnerships appears to be due to health system factors that reinforce siloed services, creating gaps in continuity and coordination. White and colleagues highlight existing power imbalances between primary care and HSS [32], which acts as another barrier to intersectoral collaboration. Despite these challenges, primary care needs to connect with HSS in the care of older adults. HSS can help address long-term conditions, support lifestyle changes, and improve the management of chronic diseases in ways that that primary care cannot do alone [2, 33].

Physicians tend to link with HSS by providing patients with community information, initiating linkages for those in need, and encouraging families to access services for their loved ones [34]. Our results identified strategies for primary care and CBHSS to build bridges to address the need-service gap for older adults including: a) using a person-focused approach; b) applying effective case coordination; c) adapting services to address access barriers or filling gaps; d) employing effective communication strategies between sectors; e) building trust and rapport between them; and (f) advocating for improved program access.

Primary care providers linked to HSS when they had previous trusted relationships. Inter-organizational trust has been studied extensively in successful inter-organizational collaborations [35, 36]. A social network analysis involving primary care and HSS showed that when there was trust in an organization’s competence, there was increased desire to collaborate [37]. Some primary care providers in our study built trust by asking for HSS endorsements from patients, sharing consult notes, and relying on staff with previous experiences with HSS. Others have found that physicians often relied on team members’ knowledge and expertise, especially if they had delegated responsibility to link to outside resources [4]. In addition, primary care physicians experience challenges, such as insufficient time and staffing to link individuals with social services [38, 39]. Interprofessional teams, practice nurses, and case managers have been shown to facilitate primary care and community linkages for older adults and others with long-term conditions, such as social isolation [2, 3, 34, 39, 40].

Although some have suggested that primary care stays up to date with resources, and agencies make efforts to increase primary care awareness of their services [41], other options may be needed given the time limitations as expressed in this study. Isaacs and colleagues reported that certain organizations act as brokers to build linkages among trusted networks of health and community services, although brokers change depending on needs being addressed [42]. Knowing which organizations are trusted brokers for which issues may help to build bridges between organizations and reduce the burden of staying up to date with all available CBHSS.

One strategy to address primary care’s lack of trust in HSS was to build stronger partnerships. However, each sector wants the other to do a little bit more: e.g., HSS providers want primary care to do more outreach, care coordination, and proactive preventative care. Further, primary care providers seemed unsure of their role in system navigation. A scoping review of system navigation in primary care explored navigation models demonstrating weaker and stronger primary care and HSS linkages [43]. A concerted effort is needed to ensure that the system navigation function is fulfilled [41]. But by who, how it is fulfilled, and under which circumstances remains in debate. A panel on patient navigation found that patients rejected the idea of a separate provider being involved [44]. However, if primary care and HSS providers do not take on system navigation functions, the burden rests on patients and families. May and colleagues suggest that the management of multimorbidity and demands for patient self-care and preventive practice result in an important shift in accountability:

… adding the *burden of treatment* to the burden of symptoms, as patients experience new and growing demands to organize and coordinate their own care … [45] (p.2).

Overall, there is a need to further evaluate navigation roles in models involving community and primary care [4], and recommend system improvements to support system navigation [41].

Although this study involved a range of primary care and HSS providers’ disciplines and roles, transferring results are limited to similar contexts. Our study involved primary care team-based clinics, and HSS agencies in a large urban Canadian setting. While our results may be limited to apply to similar settings, they are generally supported in the literature. Furthermore, most themes were raised in all or most focus groups indicating that saturation was reached. There was also some potential for social desirability bias wherein providers may have wanted to provide favorable responses. To reduce this potential, we separated primary care providers from community service providers in focus groups to encourage open and honest answers.

This study focused on gaining a deeper understanding of providers’ perspectives regarding links between primary care and HSS and system navigation. Although we identified challenges that primary care providers and community service providers experienced in working together, there remains a gap in understanding the reasons for and individual and structural causes of these weaknesses. In addition, we did not include older adults’ views on barriers to accessing services or working together with HSS as an integrated team. Co-design of programs or services requires input from all stakeholders including consumers [46]. This topic also warrants further study.

## Conclusion

Community-living older adults with MCC experience needs that could be addressed, at least in part, by integrated primary care and HSS services. However, there are many barriers to accessing the complex system of services, some of which are focused on the very relationship between primary care and HSS providers. Study participants described several strategies that may be effective in addressing system navigation barriers experienced by older adults. However, much needs to be done to create a well-coordinated, integrated system that supports not only optimal aging of older adults, but collaborative working relationships of primary care and HSS providers. Further research is needed to identify which approaches are most effective.

## Supplementary information

**Additional file 1.** Focus Group Guide

## Data Availability

Data are not publicly available due to the lack of consent from participants to share the data beyond this study.

## Abbreviations

HSS: Community-based health and social services
MCC: Multiple chronic conditions
