# Supplementary material for: Disconnected relationships between primary care and community-based health and social services and system navigation for older adults: a qualitative descriptive study
Source: BMC Fam Pract. 2020 Apr 23;21:69. doi: 10.1186/s12875-020-01143-8 (PMC7181491; doi:10.1186/s12875-020-01143-8)
Supplement: Supplementary file 1 — Additional file 1. Focus Group Guide [file 12875_2020_1143_MOESM1_ESM.docx]

**Supplementary File 1**

**Focus Group Guide**

1. What do you find are the most common health and social service needs identified by older adults living in the community and who receive primary health care?

2. What do you find are the most challenging health and social service concerns/needs to address? (for older adults living in the community and who receive primary health care)?

3. Thinking about the needs and concerns discussed so far, which two of these areas would be worth focussing on to create better clinic-community linkages? Which two would be the most challenging and why?

4. What strategies/mechanisms do you and your organization use to build credible linkages with primary care to support the health and well-being of older adults living in the community? Probe: What strategies/mechanisms are used to sustain credible linkages? Prompt: Which disciplines play key roles in building and maintaining these networks? (e.g. nurses, social workers)
